# Comparative evaluation of 3D-printed and conventional implants in vivo: a quantitative microcomputed tomographic and histomorphometric analysis

**DOI:** 10.1038/s41598-023-48315-x

**Published:** 2023-11-29

**Authors:** Hyemee Suh, Dongseob Lee, Jungwon Lee, Yang-Jo Seol, Yong-Moo Lee, Ki-Tae Koo

**Affiliations:** 1 Department of Periodontology, School of Dentistry and Dental Research Institute, Seoul National University, 101 Daehak-ro, Jongno-gu, Seoul, 03080 Republic of Korea; 2 Department of Periodontology, Seoul National University Dental Hospital, Seoul, 03080 Republic of Korea; 3 National Dental Care Center for Persons with Special Needs, Seoul National University Dental Hospital, Seoul, 03080 Republic of Korea; 4 One-Stop Specialty Center, Seoul National University Dental Hospital, Seoul, 03080 Republic of Korea

**Keywords:** Medical research, Preclinical research

## Abstract

In recent years, 3D-printing technology to fabricate dental implants has garnered widespread attention due to its patient-specific customizability and cost-effectiveness. This preclinical animal study analyzed the radiographic and histomorphometric outcomes of 3D-printed implants (3DIs) placed immediately after extraction and compared them to conventional implants (CIs). 3DIs and CIs of the same dimensions placed immediately were analyzed at 2, 6, and 12 weeks. The micro-computed tomography (micro-CT) analysis revealed statistically significant differences at 2 weeks in favor of 3DIs over the CIs in terms of bone volume/tissue volume (BV/TV), bone surface/bone volume (BS/BV), trabecular bone pattern factor (Tb.Pf), and structure model index (SMI). At 2 weeks, the mean bone-to-implant contact (BIC) of the 3DIs was greater than that of the CIs; the mean bone area fraction occupancy (BAFO) and the number of Haversian canals of the 3DIs showed no statistically significant differences compared to CIs at 2 weeks. At 6 and 12 weeks, there were no statistically significant differences between the 3DIs and CIs in any parameters. Within limitations, in the early stage of extraction socket healing, the 3DIs demonstrated a higher BIC than the CIs, presenting that 3DIs may be a potential option for immediate placement to enhance osseointegration.

## Introduction

Because of recent advances in implant therapy, dental implants are currently regarded as highly predictable treatment options for edentulism^1^. Thus, there has been increasing demand for the further improvement and optimization of implant materials and treatment protocols that would guarantee a better success rate and shorten the treatment time^2, 3^.

The conventional production of dental implants involves machining of the titanium implant rods, followed by coating or surface modification of the titanium implant surfaces to create favorable surface characteristics and enhance osseointegration potential^4^. In recent years, alternative modes of implant production have been explored. Additive manufacturing (3D printing) techniques have been investigated for their potential to replicate complex geometric designs in cost-effective ways^5^.

While conventional implants (CIs) have a limited selection of shapes and sizes and require the drilling of residual bone in cylindrical shapes to fit the shape of the implant fixture, 3D printing potentially enables the tailored production of personalized implant rods that exactly fit the shape of the tooth roots to be replaced. Prior to surgery, information obtained from cone-beam computed tomography (CBCT) imaging can be utilized as a blueprint to fabricate the customized implants. These can then be placed immediately into extraction sockets without the need for patients to wait months for bone regeneration before implant placement^6^.

Among the numerous 3D-printing technologies, selective laser melting (SLM) has recently garnered much attention^7, 8^. With this technique, implants are additively manufactured with metal powder, which is fused layer-by-layer in thin metal sheets. Contrary to conventional milling methods, in which titanium rods are subjected to subtractive processes to create the final implant form, SLM offers the benefit of less material waste as implants are printed layer-by-layer. This allows delicate control of the shape and texture, as well as greater leverage over the distribution of material. As a result, there is a significant economic advantage as there is no need for prefabricated molds or extensive tool requirements, and implants may be produced on demand following the specific needs of the patient. Furthermore, complex designs suited to the patient’s individual oral condition can be made using information obtained by computer-aided design, and accordingly fabricated by computer-aided manufacturing^9^. As such, the digital workflow expedites the production process of personalized implants, eliminating the need for external manufacturing and transportation. In addition, there is a concomitant reduction in the cost of traditional dental impressions and the added convenience for the patient.

One of the significant mechanical advantages of 3D printing is that titanium implants can be constructed with a gradient of porosity perpendicular to the long axis of the implant^10^. With this functional gradient, the inner core is denser and the outer portion is more porous; thus, the outer surface of the implant has an elastic modulus closer to that of the surrounding bone, which leads to a more natural transfer of loading stress^11^.

Furthermore, considerable emphasis has been given to the implant surface treatment, as sufficiently rough surfaces have been shown to promote osteogenic differentiation and osseointegration following implant surgery^12^. In this regard, 3D-printed implants (3DIs) are characterized by inherently rough surfaces that may facilitate the process of osteogenic differentiation and the osseointegration of implants to the surrounding bone^13^. In addition, 3D printing enables enhanced leverage in controlling the material composition. This enables control of implant porosity, size, and shape, as well as a substantial amount of pore interconnections, a characteristic that is necessary for bone ingrowth into implant surfaces^11^. While control of implant porosity is important in the establishment of secondary biological stability by ensuring uniform cell distribution, cell survival, and proliferation, this factor is often difficult to achieve with the conventional machining methods of implant manufacturing^14^.

Numerous studies of 3DIs have shown favorable clinical outcomes in vivo and have indicated a demand for further investigations into the actual implementation of 3DIs^15–18^. Previously, our group established a digital workflow for the fabrication of double-root 3DIs that were designed in the shape of lattice- and solid-type roots, and found that the macrodesign of the implants may affect the secondary stability in vivo^15^. Furthermore, another study performed by our group comparing single and double-root 3DIs showed comparable implant stability and peri-implant bone remodeling, whereas higher marginal bone loss was observed in the furcation areas of double-root 3DIs^18^. However, there have been few related studies that fundamentally compared the in vivo clinical outcomes of 3DIs and CIs.

Therefore, the present study compared the in vivo application of 3DIs and CIs that were placed immediately after extraction in dog mandibles and assessed the peri-implant radiological and histomorphometric changes after bone healing.

## Methods

### Sample size estimation

The sample size calculation was conducted based on a study by Antunes et al.^19^ with G*Power software (Heinrich-Heine-Universität, Düsseldorf, Germany). In this study, a difference in mean bone-to-implant contact (BIC) of 15 between the two groups (3DIs and CIs) was regarded as statistically significant. The standard deviation was set at 8% (effect size: 1.875). The α and β values were set at 0.05 and 0.8, respectively. A six-animal statistical unit was needed to compare two groups.

### Ethical statement

This experimental procedure was approved by the Institutional Animal Care and Use Committee of Seoul National University (No. SNU-210317-6) and followed the Animal Research: Reporting of In Vivo Experiments (ARRIVE) guidelines^20^.

### Study design

**Figure 1:**
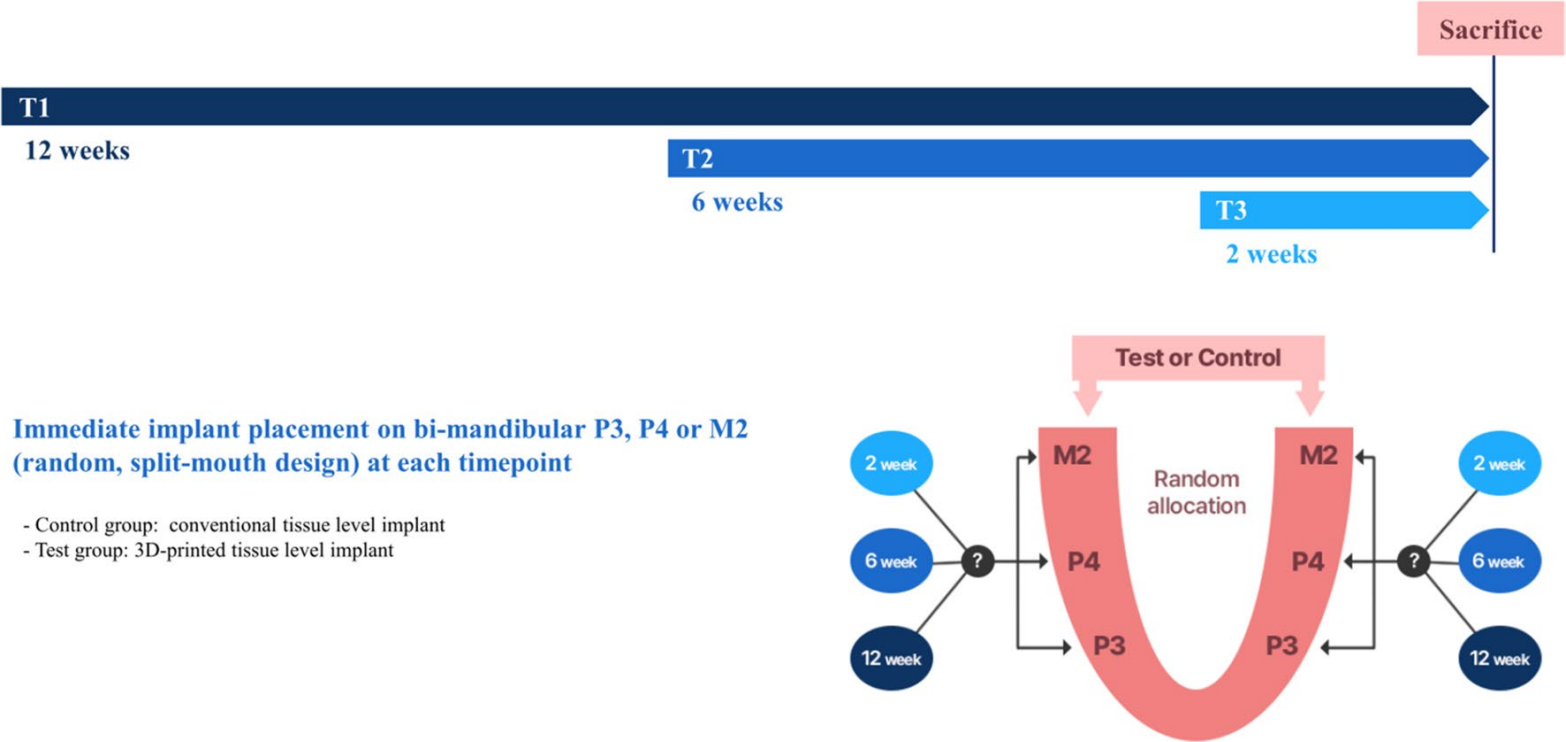
Study timeline and schematic diagram. Immediate implant placements were performed on the third premolar, fourth premolar, or second molar (P3, P4, or M2) in both mandibles, using a CI or a 3DI, based on a split-mouth design at each time point (2-, 6-, and 12-week healing periods). *CI* conventional implants, *3DI* 3D-printed implants.

The timeline and experimental design of this study are presented in Fig. 1. Six male beagle dogs, weighing approximately 10 to 15 kg and aged approximately 1 year, were included. Immediate implant placement was conducted on the mandibular third premolar, fourth premolar, or second molar (P3, P4 or M2) using a 3DI (test) or a CI (control), based on a split-mouth design at each time point (2, 6, and 12 weeks before sacrifice). In each dog, six implants (three CIs and three 3DIs) were placed. Implant placement was performed at three different time points to observe the time effect on the differences in bone healing. To control for the effect of implant position in the mandibular arch, in half of the subjects the test group was designated on the right side, and in the other half the left side. The exact position of the implant placement in terms of P3, P4, or M2 within the respective side of the arch was randomly allocated by generating a random number sequence.

### Fabrication of 3DIs

The fabrication of the 3DIs followed the workflow established in previous studies by our group^15, 21^. According to a previously established reference database regarding average tooth sizes, a standard model representative of the average dimensions of the corresponding tooth was constructed. Referring to this model, conventional tissue-level implants (Bright™, Dentium Co., Ltd., Gyeonggi-do, South Korea) with dimensions of 3.8 mm (platform diameter) × 9.0 mm (length) × 3.5 mm (apical diameter) were chosen for use in this study. The 3DIs were fabricated with the same dimensions by the SLM method with Ti–6A–l4V alloy powder using a 3D-printer (Rainbow Metal Printer™, Dentium Co., Ltd., Gyeonggi-do, South Korea). Surface modification was done by the sandblasted, large-grit, and acid-etching (SLA) method. The final products were sterilized (according to ISO 1137) using gamma-ray irradiation, a short-wavelength light emitted from a cobalt-60 (^60^Co) radioactive isotope.

### Surgical procedures

**Figure 2:**
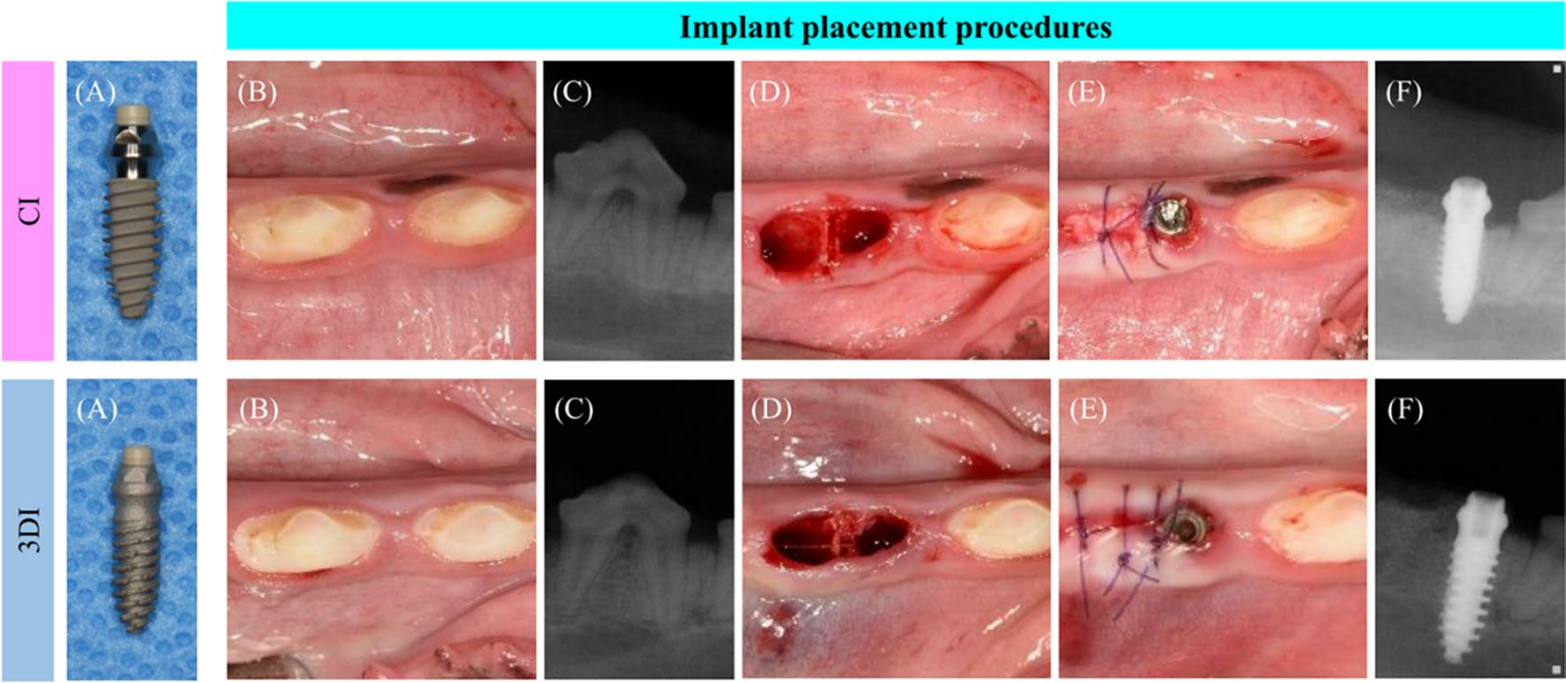
Representative clinical photographs and periapical radiographic images before and after immediate implant placement. CI and 3DI fixtures (**A**) were prepared for an immediate implant placement procedure in dog mandibles. Clinical photographs (**B**: before surgery, **D**: after extraction, **E**: after implant placement) and radiographical images (**C**: before surgeryp, **F**: after implant placement) were taken before and after the surgical procedure.

Immediate implant placement was performed on mandibular P3, P4, and M2 at each time point. Under general anesthesia induced by intravenous injection of a combination of xylazine hydrochloride (2.33 mg/kg), tiletamine/zolazepam (5 mg/kg), and atropine sulfate hydrate (0.05 mg/kg), local anesthesia was applied using 2% lidocaine HCl (1:100,000 epinephrine) before the surgical procedure. Hemisections of the same selected teeth (P3, P4 or M2) on the mandible were performed using a rotatory instrument. The teeth were luxated and extracted meticulously using an elevator and forceps. Using a Bright™ surgical drilling kit (Dentium Co., Ltd., Gyeonggi-do, South Korea), the implant site was prepared by initially drilling with a 2.5 mm drill, followed by 3.0 mm and 3.5 mm drills. Implant placement was then performed in the mesial extraction socket of the control group (CI) or the test group (3DI). The surgical site was sutured with 5-0 Monosyn. After the procedures, antibiotics (cefazolin, 20 mg/kg) and analgesics (tramadol HCl, 5 mg/kg) were injected intravenously to relieve postoperative pain and inflammation. The same surgical procedure was performed at 6 and 10 weeks on the remaining teeth. Clinical photographs and periapical radiographs were obtained with every surgical procedure (Fig. 2).

### Micro-CT analysis

The animals were sacrificed 12 weeks after the first implant placement by carotid injection with potassium chloride (75 mg/kg; Jeil Pharmaceutical Co., Ltd., Daeju, South Korea). Block biopsy samples of the implant placement sites were prepared for micro-CT and subsequent histological analyses. For micro-CT, the blocks were scanned at a 13.3 μm resolution, 60 kV energy, and 167 μA intensity using a 0.5-mm aluminum filter and a three-dimensional micro-CT machine (Bruker Skyscan1172, Kontich, Belgium). The two-dimensional images were saved as .bmp files (2240 × 2240 pixels) and followed by reorienting the axes of the sagittal and transverse images for analyses using software (Dataviewer 1.5.2.4 64-bit version, Bruker, Kontich, Belgium. The 3D data were analyzed with CTAn software (Bruker micro-CT, Kontich, Belgium). In accordance with a previous study^15^, the volume of interest (VOI) was set to a 190 μm circular band, stretching 60–250 μm from the implant surface. Within the VOI, the bone volume/tissue volume (BV/TV), bone surface/bone volume (BS/BV), trabecular bone pattern factor (Tb.Pf), and structure model index (SMI) values of the CIs and the 3DIs were compared.

### Histological processing

The histological samples were prepared by submerging the block biopsies in a fixative solution containing 10% neutral formalin buffer (Sigma-Aldrich, St. Louis, Missouri, USA) for 1 week, then dehydrated by exposing the samples to increasing grades of ethanol solutions. The samples were then embedded in methacrylate (Technovit 7200, Heraeus Kulzer, Hanau, Germany) according to the manufacturer’s instructions. The prepared samples were sectioned in a mesiodistal direction using a diamond saw and grinding system (KULZER EXAKT 400CS, Norderstedt, Germany). The final tissue sections were polished to approximately 45 ± 5 μm thickness before staining with Goldner trichrome.

### Histological and histomorphometric analysis

The prepared histological slides were examined under a light microscope (Olympus BX51, Olympus Corp., Tokyo, Japan) connected to a camera with a charged-couple device (CCD) (SPOT Insight 2 Mp scientific CCD digital camera system, Diagnostic Instruments, Inc., Sterling Heights, MI, USA) and an adaptor (U-CMA3, Olympus Corp., Tokyo, Japan). The histological images were captured as digital image files for histomorphometric analysis after scanning with the Panoramic 250 Flash III (3DHISTECH Ltd., Budapest, Hungary). Using computer-aided slide image analysis software (CaseViewer 2.2; 3DHISTECH Ltd., Budapest, Hungary), the region of interest (ROI) was set to the area encompassed by 3 implant threads in the middle portion of the implant fixture. Histomorphometric measurements were performed twice by an experienced examiner using image analysis software (ImageJ version 1.53a, National Institutes of Health, Bethesda, Maryland, USA). Within the ROI, the bone-to-implant contact (BIC) and bone area fraction occupancy (BAFO) were measured for the CIs and 3DIs. Histological observation was made at × 5 magnification, and histomorphometric measurements were performed at × 10 magnification. The mean number of Haversian canals within the ROI was counted and divided by the total area of the interthread space, delimited by an imaginary line drawn between the thread tips.

### Statistical analysis

Statistical analysis was performed using commercially available software (GraphPad Prism; San Diego, CA, USA). All numerical data were presented as means ± standard deviations. The Shapiro–Wilk test was used to assess the normality of the data distribution. The paired t-test was performed for comparisons of micro-CT findings between two groups that were placed in counter-position. The paired t-test was used for comparisons between two groups at each time point, and repeated-measures analysis of variance was used for intragroup comparisons among the three time points. Post-hoc multiple comparison was done using the Tukey test. When a normal distribution was not observed, the Wilcoxon signed rank test was performed for group comparisons. Differences were considered statistically significant at *p* < 0.05.

### Ethics approval

This study was performed under the ethical approval of the Institutional Animal Care and Use Committee of Seoul National University (No. SNU-210317-6). All methods in this animal experiment were performed in conformity with the principles of the 3R (Replacement, Reduction, and Refinement) and two major laws in Korea which are the Animal Protection Act established by the Ministry of Agriculture Food and Rural Affairs, and the Laboratory Animal Act established by the Ministry of Food and Drug Safety.Please note we have moved the section ‘Ethics approval’ to the end of the methods, as per house style.O.K. 

## Results

### Micro-CT analysis

**Figure 3:**
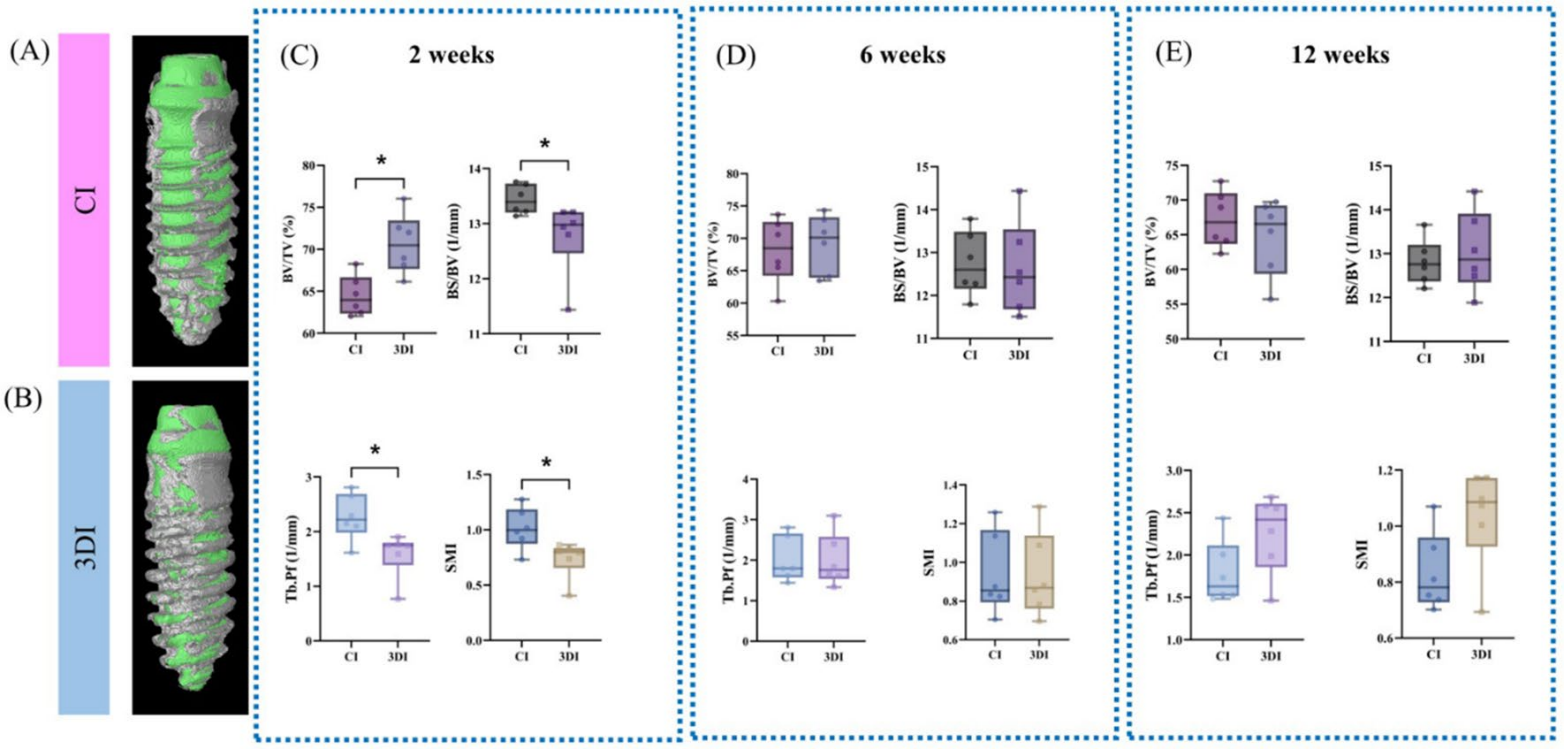
Representative microcomputed tomography (micro-CT) images of (**A**) CI and (**B**) 3DI and analyses (**C–E**). (**C**) Outcomes of micro-CT analysis at 2 weeks. (**D**) Outcomes of micro-CT analysis at 6 weeks. (**E**) Outcomes of micro-CT analysis at 12 weeks. *p < 0.05. *BV/TV* bone volume/tissue volume, *BS/BV* bone surface/bone volume, *Tb.Pf* trabecular bone pattern factor, *SMI* structure model index.

Micro-CT analysis was performed for CIs and 3DIs at 2, 6, and 12 weeks, as shown in Fig. 3.

At 2 weeks, the BV/TV values of the CIs and 3DIs were 64.47% ± 2.40% and 70.64% ± 3.58%, the BS/BV values were 13.44 ± 0.27 mm^−1^ and 12.77 ± 0.67 mm^−1^, the Tb.Pf values were 2.27 ± 0.43 mm^−1^ and 1.59 ± 0.41 mm^−1^, and the SMI values were 1.01 ± 0.19 and 0.74 ± 0.17, respectively. At 6 weeks, the BV/TV values of the CIs and 3DIs were 68.11% ± 5.00% and 69.17% ± 4.53%, the BS/BV values were 12.74 ± 0.75 mm^−1^ and 12.63 ± 1.08 mm^−1^, the Tb.Pf values were 2.01 ± 0.56 mm^−1^ and 2.00 ± 0.65 mm^−1^, and the SMI values were 0.94 ± 0.21 and 0.93 ± 0.22, respectively. At 12 weeks, the BV/TV values of the CIs and 3DIs were 67.19% ± 4.10% and 64.69% ± 5.49%, the BS/BV values were 12.81 ± 0.51 mm^−1^ and 13.05 ± 0.91 mm^−1^, the Tb.Pf values were 1.79 ± 0.37 mm^−1^ and 2.26 ± 0.47 mm^−1^, and the SMI values were 0.83 ± 0.14 and 1.04 ± 0.18, respectively.

At 2 weeks, statistically significant differences between CI and 3DI were observed for all micro-CT parameters. At 6 and 12 weeks, there were no statistically significant differences.

### Histological observations

**Figure 4:**
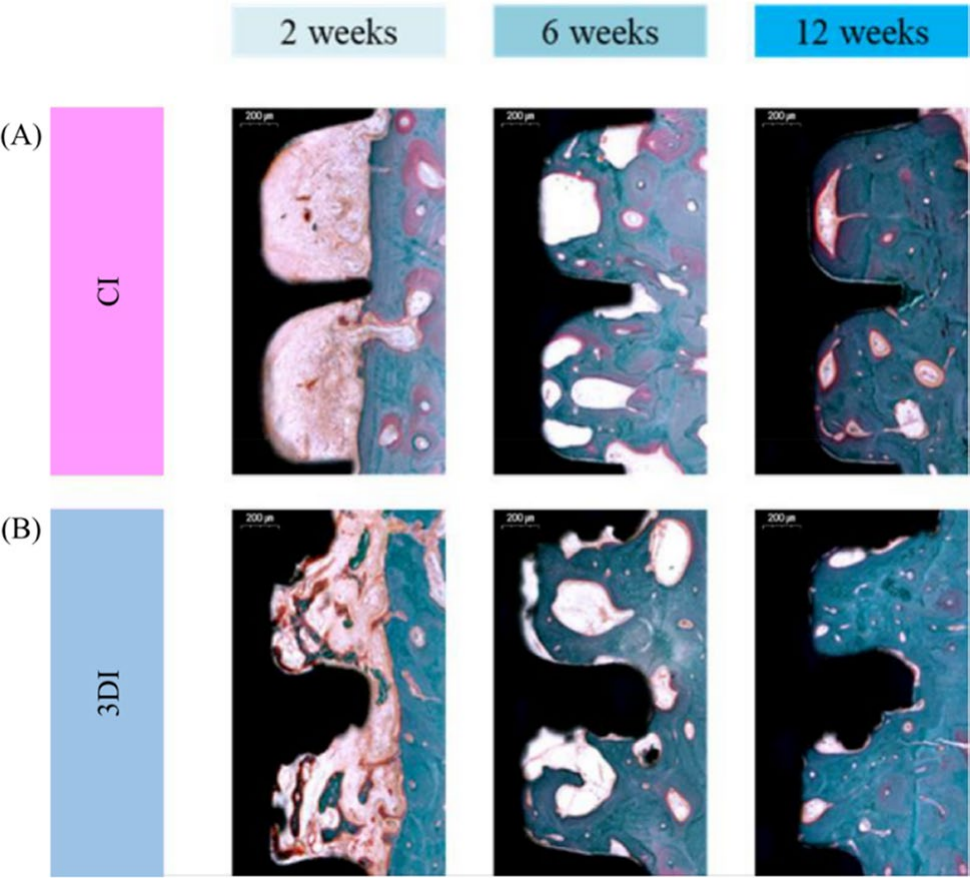
Representative histologic images of CI and 3DI at 2, 6, and 12 weeks. Histological sections of (**A**) CI and (**B**) 3DI at 2, 6, and 12 weeks after placement revealed direct bone deposition in both groups. At 2 weeks, the 3DI showed faster formation of mineralized bone deposits near the implant surface than in the CI.

Histological sections of the implants at 2, 6, and 12 weeks after placement revealed gradual new bone formation on the surfaces of both CIs and 3DIs (Fig. 4). Bone maturation and the osseointegration process were observed, in a time-dependent manner. This was highlighted by the minimal bone formation at 2 weeks after implant placement, which healed to nearly complete bone deposition on the implant surface by 12 weeks.

At 2 weeks, the presence of visibly greater formation of mineralized new bone deposits was visible near the surface of 3DI compared to CIs. By 6 and 12 weeks, both 3DI and CI showed a similar level of new bone formation.

### Histomorphometric analysis

**Figure 5:**
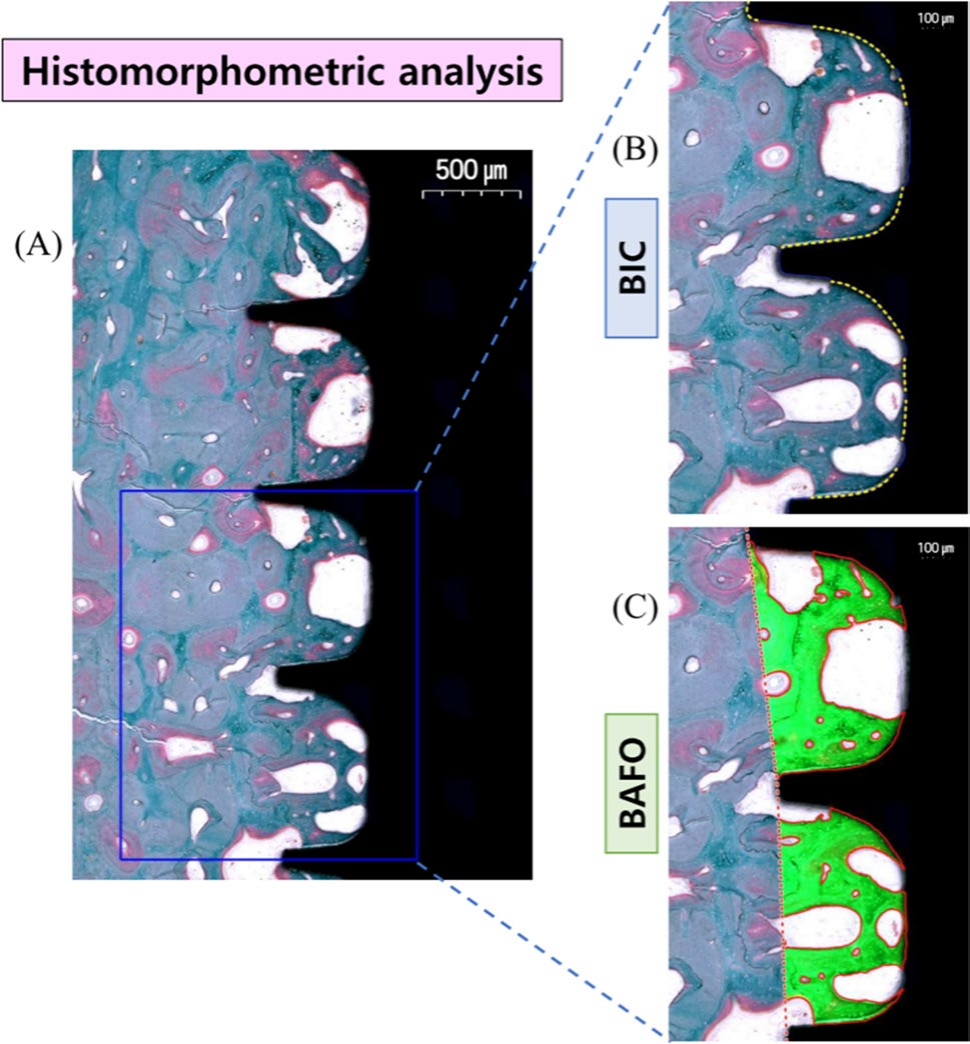
Schematic illustration of histomorphometric analysis in the ROI. (**A**) The ROI was set to the area encompassed by three implant threads in the middle portion of the implant fixture. (**B**) BIC was measured as the total length of the implant fixture in contact with bone (yellow dotted line), divided by the total length of the fixture (blue solid line). (**C**) BAFO was measured as the total area of mineralized bone (green), divided by the total area encompassed by the implant threads and an imaginary line (red dotted line) connecting the thread tips. *ROI* region of interest, *BIC* bone-to-implant contact, *BAFO* bone area fraction occupancy.

**Figure 6:**
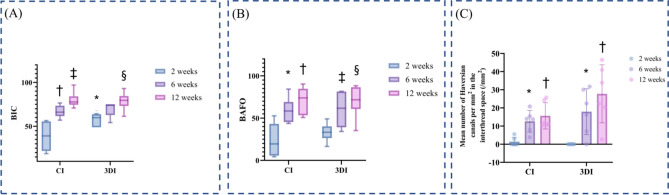
Histomorphometric analyses. (**A**) BIC of the CIs and 3DIs at 2, 6, and 12 weeks. *A statistically significant difference between the CIs and 3DIs at the same time point; ^†^statistically significant when compared with 2 weeks in the CI group; ^‡^statistically significant when compared with 2 weeks in the CI group; ^§^statistically significant when compared with 2 weeks in the 3DI group. (**B**) BAFO of the CIs and 3DIs at 2, 6, and 12 weeks. *Statistically significant when compared with 2 weeks in the CI group; ^†^statistically significant when compared with 2 weeks in the CI group; ^‡^statistically significant when compared with 2 weeks in the 3DI group; ^§^statistically significant when compared with 2 weeks in the 3DI group. (**C**) Mean number of Haversian canals per mm^2^ in the interthread space (1/mm^2^) of the CIs and 3DIs at 2, 6, and 12 weeks. *Statistically significant between 2 and 12 weeks in each CI and 3DI group; ^†^statistically significant between 2 and 12 weeks in each CI and 3DI group. *BIC* bone-to-implant contact, *BAFO* bone area fraction occupancy.

Histomorphometric analysis of the CIs and 3DIs was conducted as shown in Figs. 5 and 6. For quantitative analysis of bone formation after implant placement, the BIC and BAFO were measured within the ROI for all histological samples (Fig. 5).

The BIC values of the CIs at 2, 6, and 12 weeks were 38.60% ± 17.98%, 66.86% ± 6.96%, and 79.79% ± 8.98%, respectively (Fig. 6A). For the 3DIs, the BIC values at 2, 6, and 12 weeks were 57.55% ± 7.08%, 69.67% ± 8.37%, and 78.72% ± 10.20%, respectively. For both CIs and 3DIs, the increase in BIC with longer healing times was statistically significant (*p* = 0.003 and *p* < 0.001, respectively). At 2 weeks, the mean BIC of 3DIs was significantly higher than that of CIs (*p* = 0.024). At 6 and 12 weeks, there were no statistically significant differences in the mean BIC of the 3DIs and CIs.

The BAFO of the CIs at 2, 6, and 12 weeks were 23.73% ± 20.83%, 59.16% ± 14.85%, and 71.04% ± 15.85%, respectively (Fig. 6B). For the 3DIs, the BAFO values at 2, 6, and 12 weeks were 30.23% ± 10.60%, 60.31% ± 19.96%, and 70.42% ± 18.97%, respectively. For both CIs and 3DIs, the increasing trend of BAFO values with the passage of time was statistically significant (*p* = 0.005 and *p* = 0.001, respectively). The mean BAFO of the 3DIs and CIs showed no statistically significant differences at any time point.

The mean number of Haversian canals per square millimeter in the interthread space for CIs at 2, 6, and 12 weeks were 1.4 ± 2.3, 12.6 ± 5.9, and 15.7 ± 7.3, respectively (Fig. 6C). For 3DIs, the mean number of Haversian canals per square millimeter in the interthread space were 0.0 ± 0.0, 17.9 ± 12.63, and 27.77 ± 15.96, respectively. There were statistically significant increases in the number of Haversian canals at 6 and 12 weeks when compared with 2 weeks for both CIs (2 weeks vs. 6 weeks; *p* = 0.009, 2 weeks vs. 12 weeks; *p* = 0.001) and 3DIs (2 weeks vs. 6 weeks; *p* = 0.046, 2 weeks vs. 12 weeks; *p* = 0.003). There were no significant differences between 3DI and CI in terms of the mean number of Haversian canals at any time point.We could find a mismatch of Figure 3 data between document version and tiff version. We have followed the document version. Please confirm if the action taken is appropriate.Yes. Thanks.

## Discussion

The emergence of personalized medicine has led to a growing need for individualized care within the field of dentistry. Given the demonstrated high survival rates of implants, the current focus of attention pertains to the development of implants characterized by improved success rates. In recent times, there has been a growing interest in the additive approach as a means of producing dental implants due to its numerous potential benefits, encompassing mechanical, biological, clinical, and economic aspects.

In light of this perspective, the potential utilization of 3DIs instead of CIs is associated with a multitude of encouraging prospects. Prior studies have indicated that dental implants produced by the SLM method have the potential to yield a wide range of biological and mechanical benefits compared to conventionally machined implants. Recent studies of 3DIs have shown positive results, such as the favorable impact of the topographic roughness of 3DIs^17^. Numerous research efforts have begun to focus on the implications of 3DIs when applied in clinical settings. Some studies have fabricated 3DIs using CBCT images for immediate placement into extraction sockets, but evidence is scant and remains limited to case reports^6^. Since few studies have assessed the radiographic and histological aspects of 3D-printed titanium implants in vivo, we attempted to conduct an analysis of such considerations after surgical placement of 3D-printed titanium implants in an animal model.

In this study, immediate implant placement was conducted using 3DIs designed in accordance with a conventional tissue-level implant to provide a fundamental comparison of 3DIs and CIs while excluding the effect of the macroscopic design of the fixture on bone regeneration and remodeling. Under a split-mouth design, with either the right or left side being the test (3DIs) or control (CIs) site, implant placement was performed at the third premolar, fourth premolar, and second molar of dog mandibles. The position of the implants, each associated with a specific time point (2, 6, and 12 weeks) before sacrifice, was randomly allocated to control for the effect of bone morphology on subsequent healing related to respective positions within the dental arch (Fig. 1). Implant placement was performed at 3 different time points to observe for time-related differences in bone formation in the 3DIs and CIs. Clinical photographs and periapical X-rays (Fig. 2), taken postoperatively after every surgical procedure, showed radiographically and clinically uneventful healing in all implant positions. Therefore, all implant positions were included for micro-CT and histological analysis.

Previous studies have stressed the importance of identifying the quality of bone to characterize its biomechanical factors as it has been recognized that not only the bone mineral density, but also the morphology of the bone structure is directly related to bone toughness^22^. In this respect, micro-CT offers valuable microarchitectural information pertaining to peri-implant bone volume^23^. Furthermore, micro-CT offers 3-dimensional information which cannot be obtained with histomorphometry, in which microscopic sections are observed in 2-dimensional planes only^24^. In this study, micro-CT was used to analyze BV/TV, BS/BV, Tb.Pf, and SMI. At 2 weeks, statistically significant differences were observed between the CIs and the 3DIs for BV/TV, BS/BV. Tb.Pf, and SMI (Fig. 3). The higher BV/TV, lower BS/BV and SMI relating to more plate-like structure of trabecular bone, and lower Tb.Pf values linked with higher connectivity quantitatively indicated bone healing in favor of the 3DIs over the CIs^25–28^. By 6 and 12 weeks, there were no statistically distinct differences between the CIs and the 3DIs. The results indicated that the microstructural parameters relating to both cancellous and cortical bone, while showing differences in the early healing stage, eventually reached similar levels at the later stages of bone healing^29^. From such quantitative findings, it could be reasonably assumed that the microstructural properties of the peri-implant bone of both CIs and 3DIs show fundamental similarities, especially in the morphology and the quality of bone surrounding the CIs and the 3DIs. Whether this point relates to similar biomechanical properties under functional loading remains to be established through further studies.

After micro-CT analysis, the block biopsies underwent histological processing for qualitative and quantitative analysis. In the histological sections, the selected ROI was the area encompassed by 3 implant threads in the middle portion of the implant fixture (Fig. 5A). This area of interthread space, previously described as the “healing chamber” by Marin^30^, provides a void where a blood clot may be stabilized between the bone and implant surface and facilitate woven bone formation. As such, inspection of the implant interthread space has been used as a standard for evaluating the healing properties surrounding an implant surface. In previous studies, the number of Haversian canals within the implant interthread space was used as a parameter to assess differences in bone maturation rates^31^. In another study, the presence of well-defined osteons surrounding the Haversian canals in the bone adjacent to implant surfaces was used to establish successful osseointegration of the surface-modified implant fixtures^32^. In this study, the extent and quality of bone healing surrounding the implant fixtures were identified qualitatively by histological observation and quantitatively by measuring the total implant surface contacting bone, total mineralized bone area, and number of Haversian canals representing primary osteons within the interthread space.

Notably, in the histological analysis new bone formation seemed to visibly occur at an earlier time frame in the 3DIs than in the CIs, especially when comparing the histological sections at 2 weeks (Fig. 4). During early bone healing, abundant clusters of newly forming mineralized bone appeared and were in closer proximity to the surface of the 3DI, than in the sparsely filled area surrounding the surface of CI. This observation was consistent with previous studies, which found an increased capability of bone regeneration in 3DIs, due in part to its intrinsically rough surface characteristics^12^. A study investigating the biological properties of 3D-printed Ti–6A–4V implants showed improved adhesion, proliferation, osteogenic differentiation, and angiogenesis potential for bone marrow-derived mesenchymal stem cells when compared with conventional machined implants^33^. In vivo analysis in the same study using rat femoral condyles revealed statistically significant enhancement in the osseointegration capability of 3DIs at an early stage of healing (3 weeks). It has been speculated that the higher surface roughness of 3DIs enhances the mechanical interlocking of the surrounding bone and implants, providing better primary stability. The improved osseointegration of the 3DI surface may also help promote blood clot formation, thus boosting the osseointegration potential.

Quantitative histomorphometric analysis in terms of BIC showed a time-dependent increase in both the CIs and the 3DIs (*p* = 0.003 and *p* < 0.001, respectively) (Fig. 6A), which is consistent with the previous study^34^. At 2 weeks, the mean BIC of the 3DIs was significantly greater than that of the CIs (*p* = 0.024), but there was no statistically significant difference at other time points. Similarly, while the BAFO showed a time-dependent increase in both the CIs and the 3DIs (*p* = 0.005 and *p* = 0.001, respectively) (Fig. 6B), there were no significant differences in BAFO between the CIs and 3DIs at any time point. It is meaningful to point out that while the CIs and 3DIs showed similar osseointegration characteristics, bone regeneration around the implant surfaces occurred faster with the 3DIs, as indicated by the significantly earlier increase in the mean BIC value. Eventually, at the later stages of bone healing (6 and 12 weeks), similar levels of bone regeneration were attained in the CIs and 3DIs in terms of both BIC and BAFO. Comparison of the number of Haversian canals per mm^2^ in the interthread space also showed increasing pattern over time, revealing successful osseointegration in both groups (Fig. 6C). There was no statistically significant difference between the two groups at any time point. While showing similar histomorphometric results, the pattern of faster bone maturation associated with the 3DIs may have favorable clinical implications in implant placement during the early stage of socket healing. The possibly shortened time requirement for osseointegration may in fact accelerate the total treatment period.

In this study, the overall findings from micro-CT and histomorphometric analyses revealed that the CIs and 3DIs showed similar clinical characteristics after allowing sufficient time for newly formed bone attachment at the implant surfaces. While the 3DIs and CIs showed comparable osseointegration capabilities, an earlier pattern of bone mineralization was noted in the 3DIs both histologically and histomorphometrically. These findings support the utilization of 3DIs as a promising alternative fabrication method, since 3DIs confer many technical and clinical advantages. The ready customizability, faster osseointegration, and possibly shorter treatment time of 3DIs may present as an attractive treatment option for patients considering dental implants.

While several research studies have documented positive clinical outcomes associated with 3DIs, there is a scarcity of relevant studies that have conducted comprehensive in vivo analyses and comparisons of the clinical outcomes of CIs and 3DIs. The present study compared the in vivo application of 3DIs and CIs and assessed the histomorphometric and radiological changes around the implants following bone healing. When drawing conclusions from scientific literature, numerous influencing factors on the process of bone regeneration confound the interpretation of scientific data. As such, the present study employed a split-mouth, random allocation design to reduce the influence of individual variations, and implants of identical dimensions were utilized to facilitate a direct comparison of the bone healing surrounding conventional and 3D-printed implants. Within limitations, the findings of this research could potentially offer valuable insights that may guide future investigations regarding the clinical implementation of 3D-printed implants.

However, it must be acknowledged that the scope of this preclinical study was limited by the small number of animal subjects and the short follow-up period. Furthermore, as the main focus of this study meant to provide a fundamental comparison between CIs and 3DIs, it followed that the shape of the 3DIs was designed to be the same as the conventional cylindrical fixtures. In clinical settings, the 3DI may undergo customized prefabrication to fit the shape of the patient’s extraction socket and unique bone morphology to maximally benefit from this technology. In addition, it must be pointed out that there is a dearth of studies pertaining to the long-term mechanical durability or the fracture risks of 3DIs. Furthermore, concerns of peri-implantitis risks related to the surface roughness of 3DIs must be addressed. Future studies involving comprehensive assessments to evaluate mechanical durability and inflammation risks, greater number of subjects and randomized control trials, as well as the post-treatment evaluations of personally customized implants, may provide valuable information on the use of 3DIs in clinical environments.

## Conclusion

Within the limitations of this study, 3DIs showed comparable osseointegration characteristics to those of conventional tissue-level implants, with possible indications of faster bone mineralization near the implant surface. As such, 3DIs may be a promising alternative for immediate implant placement to enhance osseointegration during the early stages of socket healing.

## Data Availability

The datasets supporting the findings of the present study are available from the corresponding authors on reasonable request.

## Abbreviations

3DI: 3D-printed implants
CI: Conventional implants
BV/TV: Bone volume/tissue volume
BS/BV: Bone surface/bone volume
Tb.Pf: Trabecular bone pattern factor
SMI: Structure model index
BIC: Bone-to-implant contact
BAFO: Bone area fraction occupancy
CBCT: Cone-beam computed tomography
SLM: Selective laser melting
SLA: Sandblasted, large-grit, and acid-etching
ROI: Region of interest
